# Impact of elective surgery in private hospitals under the civil servant medical benefit scheme to health system

**DOI:** 10.1186/1471-2458-14-S1-O29

**Published:** 2014-01-29

**Authors:** Phatthanawilai Inmai

**Affiliations:** 1 Faculty of Medicine, Naresuan University Phitsanulok 65000, Thailand; 2 Health Insurance System Research Office (HISRO), Nonthaburi 11000, Thailand

## Background

Since May 2011, the civil servant medical benefit scheme (CSMBS) patients have been allowed to get certain elective surgeries in accredited private hospitals. The scheme pays private hospitals by case-based, Diagnosis Related Group (DRG) and the hospitals can charge extra for room and board, and doctor fee.

## Materials and methods

This study aimed to assess utilisation trend of CSMBS patients for certain elective surgeries in both public and private hospitals prior to and after policy implementation, and its impacts on service utilisation of the universal coverage scheme (UCS) members, efficiency of service provision, and burden of expenditure on patients and the scheme. Claim administrative databases of the CSMBS and UCS were employed in the analysis.

## Results

Results revealed that the number of patients receiving intra ocular lens (IOL) replacement for cataract has been prominently increased following the policy. This was due to relatively high payment rate for IOL replacement, active screening for patients with cataract in community of one private chain together with no co-payment policy and free transportation provided by the hospital. Moreover, shifting of patients to private hospitals led to the increased availability of beds in public hospitals as well as freed-up doctor’s time for UCS patients. Negative impact on public hospitals located in provinces where many patients got care from private hospitals was not found. Under the Prospective Payment System, private hospitals were more efficient in managing resources. Private hospitals tended to select providing care to better paid services; moreover, there were huge variations in co-payments paid by the patients for same procedure. The policy increased expenditure on private inpatient care; however, it did not affect overall expenditure of the scheme.

## Conclusions

In conclusion, the policy increased access to IOL replacement for cataract patients among CSMBS members; however, monitoring and evaluation system should be strengthened before scaling up the programme.

